# Multicenter prospective observational study to clarify the current status and clinical outcome in Japanese patients who have an indication for implantable cardioverter defibrillator (ICD) or wearable cardioverter defibrillator (WCD) (TRANSITION JAPAN‐ICD/WCD study): Rationale and design of a prospective, multicenter, observational, comparative study

**DOI:** 10.1002/joa3.13028

**Published:** 2024-03-27

**Authors:** Yukitoshi Ikeya, Yasuo Okumura, Rikitake Kogawa, Koichi Nagashima, Toshiko Nakai, Katsuaki Yokoyama, Kazuki Iso, Takeshi Kato, Toyonobu Tsuda, Eizo Tachibana, Satoshi Hayashida, Hidehira Fukaya, Naruya Ishizue, Hidemori Hayashi, Shunsuke Kuroda, Kazumasa Sonoda, Shiro Nakahara, Yuichi Hori, Masahide Harada, Masato Murakami, Yu‐Ki Iwasaki, Yoshiyasu Aizawa, Wataru Shimizu, Seiji Fukamizu, Mitsuru Takami, Kengo Kusano, Kohei Ishibashi, Tomoo Harada, Ikutaro Nakajima, Haruna Tabuchi, Mitsuhiro Kunimoto, Morio Shoda, Satoshi Higuchi, Itsuro Morishima, Yasunori Kanzaki, Ritsushi Kato, Yoshifumi Ikeda, Hisaki Makimoto, Tomoyuki Kabutoya, Kazuomi Kario, Takanori Arimoto, Yuichi Ninomiya, Issei Yoshimoto, Shingo Sasaki, Yusuke Kondo, Toshinori Chiba, Kennosuke Yamashita, Yosuke Mizuno, Masaru Inoue, Takeshi Ueyama, Jyunjiro Koyama, Takuo Tsurugi, Yoshiya Orita, Taku Asano, Toshiro Shinke, Kaoru Tanno, Kenta Murotani, For the TRANSITION JAPAN‐ICD/WCD study

**Affiliations:** ^1^ Division of Cardiology Nihon University Itabashi Hospital Tokyo Japan; ^2^ Department of Cardiology Nihon University Hospital Tokyo Japan; ^3^ Kanazawa University Hospital Ishikawa Japan; ^4^ Kawaguchi Municipal Medical Center Saitama Japan; ^5^ Department of Cardiovascular Medicine Kitasato University School of Medicine Kanagawa Japan; ^6^ Juntendo University Tokyo Japan; ^7^ Tokyo Rinkai Hospital Tokyo Japan; ^8^ Dokkyo Medical University Saitama Medical Center Saitama Japan; ^9^ Fujita Health University Toyoake Aichi Japan; ^10^ Department of Cardiology Shonan‐Kamakura General Hospital Kanagawa Japan; ^11^ Department of Cardiovascular Medicine Nippon Medical School Tokyo Japan; ^12^ Department of Cardiology Tokyo Metropolitan Hiroo Hospital Tokyo Japan; ^13^ Kobe University Graduate School of Medicine Hyogo Japan; ^14^ National Cerebral and Cardiovascular Center Osaka Japan; ^15^ St. Marianna University School of Medicine Hospital Kanagawa Japan; ^16^ Juntendo University Nerima Hospital Tokyo Japan; ^17^ Tokyo Women's Medical University Hospital Tokyo Japan; ^18^ Department of Cardiology Ogaki Municipal Hospital Ogaki Japan; ^19^ Department of Cardiology Saitama Medical University International Medical Center Saitama Japan; ^20^ Division of Cardiovascular Medicine, Department of Medicine Jichi Medical University School of Medicine Tochigi Japan; ^21^ Department of Cardiology, Pulmonology, and Nephrology Yamagata University School of Medicine Yamagata Japan; ^22^ Department of Cardiovascular Medicine and Hypertension Kagoshima University Graduate School of Medical and Dental Sciences Kagoshima Japan; ^23^ Division of Cardiology, and Nephrology Hirosaki University Graduate School of Medicine Hirosaki City, Aomori Japan; ^24^ Department of Cardiovascular Medicine Chiba University Graduate School of Medicine Chiba Japan; ^25^ Sendai Kousei Hospital, Heart Rhythm Center Sendai Japan; ^26^ Department of Cardiology National Hospital Organization Kanazawa Medical Center Ishikawa Japan; ^27^ Department of Cardiology Yamaguchi Prefectural Grand Medical Center Yamaguchi Japan; ^28^ Cardiovascular Center, Saiseikai Kumamoto Hospital Kumamoto Japan; ^29^ Department of Cardiovascular Center Shin‐Koga Hospital Kurume City Fukuoka Japan; ^30^ Department of Cardiology Showa University Tokyo Japan; ^31^ Department of Cardiology Showa University Koto Toyosu Hospital Tokyo Japan; ^32^ Biostatistics Center, Kurume University Kurume, Fukuoka Japan

**Keywords:** heart failure with reduced ejection fraction, implantable cardioverter defibrillator, primary prevention, wearable cardioverter defibrillator

## Abstract

**Background:**

Despite the positive impact of implantable cardioverter defibrillators (ICDs) and wearable cardioverter defibrillators (WCDs) on prognosis, their implantation is often withheld especially in Japanese heart failure patients with reduced left ventricular ejection fraction (HFrEF) who have not experienced ventricular tachycardia (VT) or ventricular fibrillation (VF) for uncertain reasons. Recent advancements in heart failure (HF) medications have significantly improved the prognosis for HFrEF. Given this context, a critical reassessment of the treatment and prognosis of ICDs and WCDs is essential, as it has the potential to reshape awareness and treatment strategies for these patients.

**Methods:**

We are initiating a prospective multicenter observational study for HFrEF patients eligible for ICD in primary and secondary prevention, and WCD, regardless of device use, including all consenting patients. Study subjects are to be enrolled from 31 participant hospitals located throughout Japan from April 1, 2023, to December 31, 2024, and each will be followed up for 1 year or more. The planned sample size is 651 cases. The primary endpoint is the rate of cardiac implantable electronic device implementation. Other endpoints include the incidence of VT/VF and sudden death, all‐cause mortality, and HF hospitalization, other events. We will collect clinical background information plus each patient's symptoms, Clinical Frailty Scale score, laboratory test results, echocardiographic and electrocardiographic parameters, and serial changes will also be secondary endpoints.

**Results:**

Not applicable.

**Conclusion:**

This study offers invaluable insights into understanding the role of ICD/WCD in Japanese HF patients in the new era of HF medication.

## Graphical abstract

The TRANSITION JAPAN‐ICD/WCD study explores the clinical outcomes of Japanese HFrEF patients meeting implantable cardioverter defibrillator (ICD) and wearable cardioverter defibrillator (WCD) criteria, including those who choose or decline device implantation, to assess the ICD/WCD current status after the introduction of new heart failure drugs like SGLT2i or ARNi in Japan.

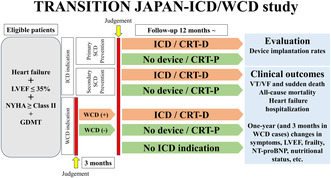

## 1 INTRODUCTION

The effectiveness of implantable cardioverter defibrillator (ICD) has been well established for both secondary prevention who experienced ventricular fibrillation/ventricular tachycardia (VF/VT), and primary prevention of sudden cardiac death (SCD) in patients with reduced left ventricular ejection fraction (HFrEF). Guidelines from organizations such as the Japanese Circulation Society (JCS), the American Heart Association (AHA), the American College of Cardiology (ACC), the Heart Rhythm Society (HRS), and the European Society of Cardiology (ESC) recommend ICD implantation at class IIa or higher for eligible patients.^1^, ^2^, ^3^ Additionally, for HFrEF patients with a wide QRS on their electrocardiogram and left ventricular ejection fraction (LVEF) less than 35%, the prognostic impact of cardiac resynchronization therapy pacemaker (CRT‐P) and CRT defibrillator (CRT‐D) has become evident^4^, ^5^, ^6^, leading to similar recommendations of class IIa or higher for implantation as outlined in the guidelines of organizations such as JCS, AHA, ACC, the Heart Failure Society of America (HFSA), and ESC.^1^, ^7^, ^8^

Nevertheless, underuse of ICD has been reported especially in Asia including Japan.^9^, ^10^ Several factors contribute to this underutilization, including physicians' awareness, patients' consciousness, and various social factors, such as those associated with aging and economic considerations in Japan.^11^, ^12^, ^13^, ^14^, ^15^, ^16^ However, in recent years, studies undertaken in Japan, such as the HINODE trial, have shown mortality rates and rates of ventricular arrhythmias as high as those in Western countries.^17^ It has attracted attention to the potential significance of ICD/CRT‐D implantation as a primary prevention in Japan.

In recent years, new standard medications such as angiotensin‐receptor‐neprilysin inhibitor (ARNi) and sodium‐glucose cotransporter 2 inhibitor (SGLT2i) also reduce fatal arrhythmias and SCD in heart failure (HF) patients.^18^, ^19^ Furthermore, the effectiveness of wearable cardioverter defibrillator (WCD) has been demonstrated in high‐risk patients with structural heart disease who do not meet the criteria for ICD implantation (within the first 3 months after starting drug therapy).^20^, ^21^ However, the use of WCD also has not gained widespread acceptance in Japan. The evidence for ICD, CRT‐P, CRT‐D, and WCD was established in an era when there were no new HF standard medications. Based on the accumulation of evidence in Japanese people after the introduction of new HF medications, we believe it is a timely moment to reevaluate the significance of using these devices for HFrEF cases and high‐risk cases of VF/VT. Therefore, the purpose of this study is (1) to investigate the real‐world device implantation rates in HFrEF patients who meet the criteria for receiving an implantable device, including ICD, CRT‐D, CRT‐P, and WCD and (2) to compare characteristics and clinical outcomes between patients with and without device implantation.

## 2 METHODS

### 2.1 Study design and setting

**FIGURE 1:**
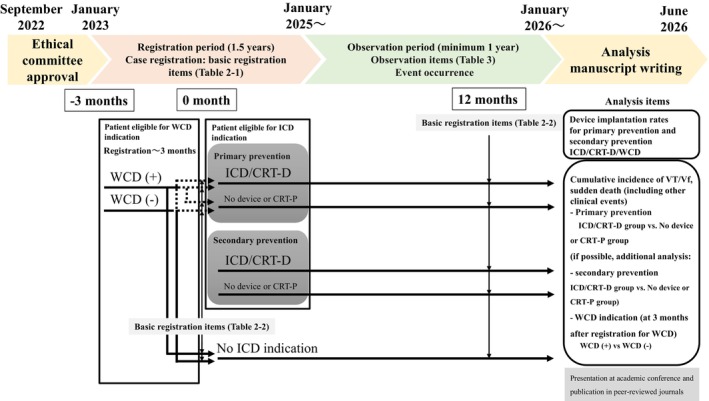
Flow diagram of the TRANSITION JAPAN‐ICD/WCD study.

The study will be based on TRANSITION JAPAN‐ICD/WCD, a large‐scale, multicenter, prospective registry including three types of HFrEF patients eligible for ICD for primary or secondary prevention of SCD, or WCD in Japan. Enrollment began on April 1, 2023, and the inclusion is planned to end on December 31, 2024, with all patients enrolled being followed up for at least 1 year (with the final follow‐up occurring on or before December 31, 2025). 31 institutions in Japan are participating in the registration (supplemental Figure S1). A flow diagram illustrating the overall follow‐up period and data analysis for the TRANSITION JAPAN‐ICD/WCD study is presented in Figure 1.

### 2.2 Eligibility and non‐eligibility

This study focuses on HFrEF patients who meet the criteria for receiving ICD for primary and secondary prevention of SCD and those who meet the criteria for WCD for primary prevention. Participants must be 20 years of age or older at the time of obtaining consent and provide written consent based on their own free will after receiving sufficient explanation regarding participation in this study. Patients eligible for this study will be basically fulfilled with the criteria of class I or IIa for ICD including sub‐cutaneous ICD (*S*‐*ICD*), and WCD as outlined in the JCS guidelines^1^: (1) patients with coronary artery disease (CAD) (≥40 days after myocardial infarction (MI) and at least 90 days after revascularization) or non‐ischemic cardiomyopathy who have NYHA class II or greater symptoms and an LVEF ≤35% despite optimal medical therapy, irrespective of whether they've experienced NSVT (criteria for primary prevention of SCD), (2) CAD or non‐ischemic cardiomyopathy who have NYHA class II or greater symptoms and an LVEF ≤35% accompanied by ventricular fibrillation (VF) or out‐of‐*hospital* cardiac *arrest* requiring electrical shock, sustained VT or unknown syncope, not due to reversible causes (criteria for secondary prevention of SCD), and (3) patients with LVEF ≤35% and HF symptoms of NYHA class II or III, and within 40 days after the onset of acute MI, and are within 90 days after coronary artery bypass or percutaneous coronary intervention, or within 90 days after the acute onset of HF due to a non‐ischemic etiology (WCD criteria). Therefore, this study will also include the patients who have opted not to receive ICD or WCD based on the patients' preference, including those without any cardiac device or those with CRT‐P. The following patients are excluded from this study: (1) those with a life expectancy of less than 12 months, (2) patients with frequent recurrent VT or VF that cannot be controlled with antiarrhythmic drugs or catheter ablation, (3) severe HF patients classified as NYHA class IV, (4) individuals unable to provide consent for the study, and (5) others judged unsuitable as study subjects by the principal researcher.

In short, the target cases for this study are those aged 20 or older with LVEF ≤35% and NYHA class II or higher HF symptoms despite adequate medical therapy, who do not fall under the exclusion criteria mentioned above.

### 2.3 Study schedule

Patients diagnosed as eligible for ICD or WCD indications will start enrollment in this study when informed consent regarding their medical condition and treatment options is obtained. All items at the time of enrollment and follow‐up in both of patient groups eligible for ICD and WCD indications, are listed in Tables 1 and 2. The brief items include age, gender, height, weight, dairy habits, underlying heart disease, NYHA functional classification, medical history, Clinical Frailty Scale,^22^ medication history including HF treatment, the presence of atrial fibrillation/flutter or NSVT, history of revascularization or ablation for NSVT/VT, history of cancer, hemodialysis, echocardiographic parameters, simple electrocardiogram information, and laboratory data. For patient eligible for ICD indications, follow‐up items including laboratory data, echocardiographic and electrocardiographic parameters, NYHA classifications, Clinical Frailty Scale scores, and HF medications will also be collected again at 12 months after enrollment. For patients eligible for WCD indications, we will collect these follow‐up items at 3 months after enrollment, and again at 12 months thereafter. Simultaneously, we will monitor the observation items for at least 1 year, and until the final follow‐up period in cases followed up beyond 1 year, as in Tables 1 and 3. These include device introduction information, invasive treatments, and the occurrence of clinical events. After the observation period ending in December 2025, we will organize and analyze the data comprehensively by March 2026. Results of the study will be published in one or more peer‐reviewed journals by June 2026.

**TABLE 1:** Study schedule and main observation items.

Patient eligible for the indication of an ICD (including CRT‐D) Item(s) recorded		Registration	At 12 months after registration*	Final follow‐up
Patient information	Age, sex, height, weight, lifestyle habits, comorbidities, etc.	●		
	Electrocardiogram Echocardiogram	●	●	
	NYHA functional classification, Clinical Frailty Scale	●	●	
	Laboratory test results, medication information	●	●	
Observation items	Device implantation date (If not implanted, provide reason)	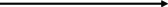		
	Invasive cardiac interventions	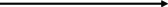		
	Clinical events (VT, VF, hospitalization for heart failure, cardiovascular death, etc.)	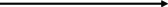		

Patient eligible for the indication of a WCD Item(s) recorded		Registration	At 3 months (corresponding to the time of WCD removal phase)	At 12 months after WCD removal phase*	Final follow‐up
Patient information	Age, sex, height, weight, lifestyle habits, comorbidities, etc.	●			
	Electrocardiogram Echocardiogram	●	●	●	
	NYHA functional classification, Clinical Frailty Scale	●	●	●	
	Laboratory test results, medication information	●	●	●	
Observation items	Device implantation date (If not implanted, provide reason)	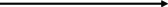			
	Invasive cardiac interventions	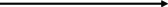			
	Clinical events (VT, VF, hospitalization for heart failure, cardiovascular death, etc.)	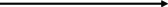			

* Allowable range: within 3 months.

Abbreviations: NYHA, New York Heart Association; VF, ventricular fibrillation; VT, ventricular fibrillation.

**TABLE 2:** Basical registration items (patient information).

(1) At registration
Date of informed consent Age, gender, height, weight Smoking habits: Current/Former/Never Alcohol consumption: Daily / 2–3 times a week/Never Underlying heart disease (Ischemic cardiomyopathy/non‐ischemic cardiomyopathy [Dilated cardiomyopathy/hypertrophic cardiomyopathy/hypertensive heart disease/others]) NYHA functional classification (I/II/III/IV) Medical history (Diabetes, hypertension, dyslipidemia, ischemic heart disease [History of myocardial infarction/Angina], history of heart failure, chronic obstructive pulmonary disease, history of stroke, peripheral arterial disease) Clinical Frailty Scale: 1–9 Medication History ① Beta‐Blockers: Yes/No ② ACEi/ARB (angiotensin‐converting enzyme inhibitor / angiotensin receptor blocker): Yes/No ③ Mineralocorticoid Receptor Antagonists: Yes/No ④ ARNi (Angiotensin Receptor‐Neprilysin Inhibitor): Yes/No ⑤ SGLT2i (Sodium‐Glucose Cotransporter 2 Inhibitors): Yes/No ⑥ Ivabradine: Yes/No ⑦ Pimobendan: Yes/No ⑧ Digoxin Preparations: Yes/No ⑨ Antiarrhythmics: Sodium Channel Blockers (Procainamide/Disopyramide/Flecainide/Cibenzoline/ Pirmenol/Aprindine/Propafenone), Potassium Channel Blockers (Amiodarone/Sotalol/Bepridil) ⑩ Anticoagulants: Warfarin/Direct Oral Anticoagulants (DOACs) ⑪ Antiplatelet Agents: Yes/No ⑫ Lipid‐Lowering Medications: Yes/No ⑬ Diabetes Medications (excluding SGLT2i): Yes/No History of revascularization (percutaneous coronary intervention, coronary artery bypass grafting) Presence of atrial fibrillation/flutter (paroxysmal/persistent/permanent) Presence of NSVT (≥5 beats) History of ablation therapy for NSVT/VT History of cancer (gastrointestinal/lung/other) Dialysis (yes/no) Echocardiogram (LVEDV [mL], LVESV [mL], LVDd [mm], LVDs [mm], LAD [mm], LVEF [%], presence of moderate or severe valvular disease [MR, TR, AR, AS]) Electrocardiogram (heart rate, rhythm, QRS duration [ms], presence of ventricular pacing) Laboratory data (NTproBNP [pg/mL] or BNP [pg/mL], WBC [×10^9^/L], lymphocyte count [×10^9^/L], Hb [g/dL], Plt [×10^9^/L], BUN [mg/dL], Cr [mg/dL], eGFR [mL/min/1.73 m^2^], UA [mg/dL], AST [U/L], ALT [U/L], rGTP [U/L], Tcho [mg/dL], Na [mmol/L], K [mmol/L], TP [g/dL], Alb [g/dL], CRP [mg/dL]) Confirmation of performance only Signal‐averaged electrocardiogram (present/absent) Holter monitoring (present/absent) Cardiac MRI (present/absent)
(2) At 12 Months (±3 Months) (WCD indication group: at 3 Months corresponding to the time of WCD removal phase and 12 months after removal phase)
NYHA functional classification (I / II / III / IV) Echocardiogram (LVEDV [mL], LVESV [mL], LVDd [mm], LVDs [mm], LAD [mm], LVEF [%], presence of moderate or severe valvular disease [MR, TR, AR, AS]) Electrocardiogram (heart rate, rhythm, QRS duration [ms], presence of ventricular pacing) Labotatory data (NTproBNP [pg/mL] or BNP [pg/mL], WBC [×10^9^/L], lymphocyte count [×10^9^/L], Hb [g/dL], Plt [×10^9^/L], BUN [mg/dL], Cr [mg/dL], eGFR [mL/min/1.73 m^2^], UA [mg/dL], AST [U/L], ALT [U/L], rGTP [U/L], Tcho [mg/dL], Na [mmol/L], K [mmol/L], TP [g/dL], Alb [g/dL], CRP [mg/dL])

Abbreviations: AR, aortic regurgitation; AS, aortic stenosis; Alb, albumin; ALT, alanine aminotransferase; AST, aspartate aminotransferase; BNP, brain natriuretic peptide; BUN, blood urea nitrogen; CRP, C‐reactive protein; Cr, creatinine; eGFR, estimated glomerular filtration rate; Hb, hemoglobin; LAD, left atrial diameter; LVDd, left ventricular diastolic diameter; LVDs, left ventricular systolic diameter; LVEF, left ventricular ejection fraction; LVEDV, left ventricular end‐diastolic volume; LVESV, left ventricular end‐systolic volume; end‐systolic volume; MR, mitral regurgitation; NT‐proBNP, N‐terminal pro‐brain natriuretic peptide; NSVT, Non‐sustained ventricular tachycardia; NYHA, New York Heart Association; Plt, platelet; rGTP, γ‐glutamyltranspeptidase; Tcho, total cholesterol; TP, total protein; TR, tricuspid regurgitation; UA, uric acid; VT, ventricular tachycardia; WBC, white cell count.

**TABLE 3:** Observation items.

Observation Items (To be observed for a minimum of 12 months from the date of registration)
Device introduction For a case who are decided to receive no device implantation: Reasons for no introduction: (1) Patient does not want risk associated with implantation. (2) Patient does not believe in benefit of ICD/CRT‐D/WCD. (3) Patient unable to pay for device. (4) Other reasons. For a case of introduction: Date of device implantation, indication for device implantation (primary prevention / secondary prevention), type of device and product name (WCD/ICD/CRT‐P/CRT‐D/other), (new implantation/upgrading) For a WCD case, the date of removal Invasive treatments during follow‐up Date of invasive treatment, type of invasive treatment (implantable cardiac device/implantable loop recorder/catheter ablation/revascularization/device reoperation) Clinical event investigation Event Date ① Presence of VT (five or more consecutive beats), VF, and/or shock events (including inappropriate shocks) ② Presence of death (cardiovascular death, other causes of death: specify details) ③ Hospitalization due to heart failure ④ Other cardiovascular events requiring hospitalization ⑤ Stroke/TIA and systemic embolism ⑥ Myocardial infarction, unstable angina ⑦ Major bleeding (ISTH criteria) ⑧ Clinically significant bleeding

Abbreviations: CRT‐D, cardiac resynchronization therapy defibrillator; CRT‐P, cardiac resynchronization therapy pacemaker; ICD, implantable cardioverter defibrillator; ISTH, International Society on Thrombosis and Haemostasis; TIA, transient ischemic attack; VT, ventricular tachycardia; VF, ventricular fibrillation; WCD, wearable cardioverter defibrillator.

### 2.4 Primary and secondary endpoints

The primary endpoint is the rate of cardiac implantable electronic device implementation. The secondary endpoints are shown in Table 4. Other significant factors besides the primary endpoint include the incidence of VT/VF, the presence of sudden death, all‐cause mortality, and HF hospitalization, as well as other variables. We will gather standard clinical background information, each patient's Clinical Frailty Scale score, laboratory test results (including measures of nutritional status), and serial changes, which will also serve as secondary endpoints.

**TABLE 4:** Primary and secondary endpoints.

Primary endpoint	
Rate of cardiac electronic device implantation (ICD, CRT‐D, WCD, CRT‐P, or None)	
Secondary endpoints	
Occurrence of any of the following	
1. VT/VF and Sudden death^a^	
2. Death from any cause	
3. Cardiovascular events (stroke/TIA or systemic embolism, myocardial infarction/unstable angina, cardiovascular death [including sudden cardiac death], hospitalization for heart failure, or other cardiovascular event requiring hospitalization)	
4. Stroke/TIA or systemic embolism	5. Myocardial infarction/unstable angina
6. Heart failure requiring hospitalization or other cardiovascular event requiring hospitalization	
7. Major bleeding (ISTH criteria^b^)	8. Clinically significant bleeding
9. Major bleeding or clinically significant bleeding	10. Cardiovascular death
11. Other causes of death	12. Device‐related complications
13. Invasive cardiac interventions (surgery, catheter‐based treatments, pacemaker implantation, etc.)	
14. If patients refused implantation, reasons for ICD/CRT‐D/WCD implantation refusal (1. Patient does not want risk associated with implantation, 2. Patient does not believe in benefit of ICD/CRT‐D/WCD, 3 Patient unable to pay for device, or 4 Other reasons)	
Change in the following from the time of enrollment to 1 year (WCD cases: at the time of WCD removal and 12 months after removal):	
1. NYHA (New York Heart Association) functional classification, Clinical Frailty Scale	
2. Echocardiographic measures	
3. Hemoglobin, hematocrit, creatinine, NT‐proBNP or BNP	
4. Nutritional status markers (serum albumin concentration, white blood cell count [peripheral blood lymphocyte count], total cholesterol, C‐reactive protein)	

Abbreviations: BNP, brain natriuretic peptide; CRT‐D, cardiac resynchronization therapy defibrillator; CRT‐P, cardiac resynchronization therapy pacemaker; ICD, implantable cardioverter defibrillator; NT‐proBNP, N‐terminal pro‐brain natriuretic peptide; TIA, transient ischemic attack; VF, ventricular fibrillation; VT, ventricular tachycardia; WCD, wearable cardioverter defibrillator.

^a^ Sudden death: natural death occurring within 24 h of the onset of acute symptoms or instantaneous death, excluding deaths resulting from external factors.

^b^ The International Society for Thrombosis and Haemostasis (ISTH) defines major bleeding as follows: fatal bleeding, bleeding into a major organ or critical area (e.g., intracranial, retroperitoneal, pericardial, intraspinal, intra‐articular, intraocular bleeding), a decrease in the hemoglobin concentration of 20 g/L or more, or transfusion of at least 2 units of blood.^23^

### 2.5 Sample size and calculation data

Last year, the number of new patients wearing WCD or undergoing ICD, CRT‐P, CRT‐D, and S‐ICD implantation at our hospital was approximately 30 cases. Therefore, we expect to register about 60 participants per year at our institution for this study (comprising 50% new device implantation cases and 50% non‐implant cases). We also collected device implantation numbers from each participating medical institution (eight institutions) on a yearly basis through a preliminary survey. We initially set the total achievable target registration number at 651 cases, comprising 50% new device implantation cases and 50% non‐implant cases, over a 1.5‐year registration period. However, due to delayed enrollment from these institutions, we expanded the study to include 23 additional institutions (a total of 31 institutions) and extended the registration period to 1.7 years to reach the target number of cases.

### 2.6 Statistical analysis

Continuous variables will be presented as mean ± SD or median (25th, 75th percentile) values and categorical variables as the number (%) of patients. Between‐group differences in continuous variables will be analyzed by unpaired *t*‐test or Mann–Whitney U‐test, and between‐group differences in categorical variables will be analyzed by chi‐squared or Fisher's exact test. Serial changes in NYHA, Clinical Frailty Scale, Echocardiographic parameters, and biomarkers will be evaluated by paired *t*‐test. We will examine device introduction rates, especially focusing on the ICD (CRT‐D) implantation as primary prevention, and compare patient characteristics between the ICD/CRT‐D group and non‐ICD (no device/CRT‐P) group. We will investigate the distribution of reasons for ICD/CRT‐D/WCD implantation refusal. Patients eligible for a WCD indication, whether they are using a WCD or not, undergo evaluation for primary prevention ICD eligibility within the first 3 months after registration. If they meet the primary prevention ICD criteria at 3 months after registration, they are categorized as those eligible for ICD primary prevention indication. However, a subset of patients does not meet these criteria and is categorized as the “no ICD indication” group. We expect that most patients will fall into this “no ICD indication” group, and we will investigate and clarify the characteristics of these patients among those eligible for a WCD indication.

Clinical events, such as VT/VF and sudden death, and cardiovascular events, for each group will be reported as percentages of the total population and annual frequencies per person‐year. Additionally, we will utilize the Kaplan–Meier method to calculate the cumulative event rates of clinical events, particularly focusing on VT/VF and sudden death, in the ICD/CRT‐D group, the non‐ICD (no device/CRT‐P) group among patients eligible for primary prevention, and compared these two groups using the log‐rank test. We will conduct Cox proportional hazards analysis to estimate hazard ratios (HRs) and 95% confidence intervals (CIs) for factors related to clinical events. Since the cardiovascular event rate is expected to provide sufficient data for multivariate analysis, we will conduct multivariate analysis to calculate the HR and 95% CI of the non‐ICD (no device/CRT‐P) group relative to the ICD/CRT‐D group in primary prevention for cardiovascular events by adjusting for clinically relevant factors. Based on the HINODE study, the annual occurrence rate of appropriate therapy for VT/VF with heart devices was approximately 6%. The all‐cause mortality rate for the group that did not have an ICD implanted (non‐device and pacing cohorts) was in the range of 8% (over the entire observation period).^17^ In the MADIT‐RIT Trial, the rate of appropriate ICD operation was also above 6%.^24^ In the MADIT II Trial, the rate of sudden cardiac death was approximately 10% in the non‐ICD group and less than 4% in the implanted ICD group, respectively.^15^, ^25^ Considering the low incidence of VT/VF and sudden death in primary prevention (approximately 5%–10%), the limited number of events may render multivariate Cox proportional hazards analysis impractical. Therefore, the propensity score model will be developed using selected variables according to clinical relevance. We will again draw the Kaplan–Meier method along with HR and 95% CI for VT/VF and sudden death for the propensity matched non‐ICD patient group compared with the propensity matched ICD patient group in primary prevention. As such, the results of elected patients after propensity score matching, along with all cohorts, along will be presented in the main text. For other groups, like those eligible for secondary prevention ICD and WCD, we plan to conduct the most comprehensive adjustment analysis possible, considering that the adjustable variables may change with the increasing number of cases.

## 3 RESULTS

### 3.1 Ethics and dissemination

The study was registered with the UMIN Clinical Trials Registry (UMIN000050180). It conforms to the Declaration of Helsinki^26^ and the Ethical Guidelines for Clinical Studies issued by the Ministry of Health, Labour and Welfare, Japan. All study participants will provide written informed consent and may withdraw their consent at any time. This study protocol has been approved by the Institutional Review Board (IRB) of Nihon University Itabashi Hospital, Clinical Research Judging Committee (IRB no: RK‐221213‐1) and/or the participating hospitals' IRBs if required.

### 3.2 Patient and public involvement statement

Neither patients nor members of the public have been or will be involved in the design of the study, its planning or the data collection or data analysis.

## 4 DISCUSSION

This multicenter observational study, based on registry data, aims to investigate ICD/WCD introduction rates and explore the reasons for device refusal in HFrEF patients eligible for ICD/WCD indications, with a particular focus on primary prevention for SCD in Japan. Unlike numerous studies on clinical outcomes in patients receiving device therapy like ICD and CRT‐D, this study is unique in its focus on HFrEF patients eligible for ICD/WCD indications, including those not receiving a device. Therefore, another study objective is to compare long‐term clinical outcomes, including VT/VF, sudden death, and other cardiovascular events, between HFrEF patients receiving the device (device introduction group) and those not using it (non‐device introduction group).

In recent years, the westernization of lifestyles and an aging population have led to a significant increase in HF patients in Japan.^27^, ^28^ While the prevention of cardiovascular deaths, including SCD, is known to improve the prognosis in HFrEF patients,^6^ there is a problem of underutilization of ICDs, as reported in previous studies,^9^, ^10^ and we also often encounter this issue in clinical practice. Recent evidence supporting the primary prevention of ICDs and WCDs for SCD has increased, even in Japan.^15^, ^16^, ^17^ This has influenced physicians' awareness of ICD/WCD indications, potentially leading to an increased rate of ICD implantation among eligible patients in Japan. On the other hand, new HF medications like ARNi, SGLT2i, and vericiguat,^18^, ^19^, ^29^ which may reduce the incidence of SCD and cardiovascular events, can also impact both our awareness of device implantation and clinical outcomes, regardless of whether patients receive device implantation. The reasons for refusing to use ICD and WCD will provide valuable insights for patient education, leading to appropriate device indications. Additionally, Japan is grappling with increased health care and caregiving costs due to its aging population, making it crucial to select patients for ICD/WCD judiciously. As seen above, we are currently in a transition period for ICD/WCD indications and understanding their prognostic role and future clinical outcomes is essential. In particular, there is limited data regarding the prognosis of HFrEF patients who meet the criteria for these devices but do not undergo implantation at this new stage. The novelty of this study lies in elucidating the current state of treatment, including new HF drugs and prognosis, in patients with HFrEF who meet the criteria for ICD/WCD, regardless of whether they actually receive these devices, in present‐day Japan. This study, which comprehensively analyzes different patient groups with a primary focus on primary prevention, will help better understand the outcomes and associated factors of their respective treatments. Japan, as the first country to experience a super‐aged society, anticipates this global demographic shift.^30^ Therefore, it is paramount to establish evidence on the role of ICD in this era, generating unique insights in the Japanese context and applying them to patient selection in clinical practice.

Despite its strengths, this observational study has limitations. It cannot establish cause‐and‐effect relationships, making it challenging to definitively determine the benefits of ICD/WCD implantation. Controlling patient bias and confounding factors, even with adjustments through multivariate analysis or matching techniques, is not always complete. Additionally, this study focuses on a single race/ethnicity.

## 5 CONCLUSION

The TRANTISION‐ICD/WCD study is a prospective multicenter registry‐based study focused on investigating real‐world ICD/WCD introduction rates among HFrEF patients eligible for ICD/WCD indications. This study will provide valuable prognostic insights into understanding the clinical outcomes, including SCD or HF hospitalization, in HFrEF patients who do or do not receive ICD/WCD.

## AUTHOR CONTRIBUTIONS

YIkey and YOk wrote the first draft of the protocol manuscript and carried the overall responsibility for the full study and the study protocol. YIkey, YOk, KYo, TKat, HF, HH, SN, and MH contributed substantially to the study concept and design, critically reviewed the manuscript and contributed to the acquisition, analysis, and interpretation of data. YOk obtained the finding. YIkey, YOk, RKo, KN, TN, KYo, KIso, TKat, TTsud, ET, SHa, HF, NI, HH, SK, KS, SN, YH, MH, MM, YIw, YA, WS, SF, MT, KKu, KIsh, TH, IN, HT, MK, MS, SHi, IM, YKa, RKa, YIked, HM, TKab, KKa, TAr, YN, IYo, SS, YKo, TC, KYa, YM, MI, TU, JK, and TTsur, YOr, TAs, TS, and KT collected the data and conducted the study, and approved the final version of this manuscript. KM provided critical comments on statistical methods and contributed to the analysis and interpretation of the data.

## CONFLICT OF INTEREST STATEMENT

YOk received research funding from Bayer Healthcare and Biosense Webster, Inc, and received scholarship grant from Boston Scientific Japan, and received speaker honoraria from Daiichi‐Sankyo, Bayer Healthcare, and Bristol‐Meyers Squibb, Ono Pharmaceutical, and Medtronic Japan, and affiliated with endowed courses from Boston Scientific Japan, Japan Lifeline, Fukuda Denshi, Abbott Medical Japan, BIOTRONIK Japan, and Medtronic Japan. RKo is affiliated with an endowed division supported by BIOTRONIK Japan, Abbott Medical Japan, Japan Lifeline and Medtronic Japan. KN received research funding from Johnson & Johnson K.K. TN received lecture fees from Abbott Medical Japan, Medtronic Japan, and BIOTRONIK Japan. TKat received lecture fees from Daiichi‐Sankyo, Bristol‐Myers Squibb, Bayer Healthcare, Boston Scientific Japan, Abbott Japan, Nihon Kohden and Medtronic Japan. HF received speaker honoraria from Daiichi‐Sankyo, Bayer Yakuhin, Nippon Boehringer Ingelheim, Johnson & Johnson K.K., Abbott Medical Japan, Medtronic Japan and Japan Lifeline. SK received research funding from Johnson & Johnson K.K. SN received speaker honoraria from Bayer Healthcare, Daiichi‐Sankyo, Bristol‐Meyers Squibb and Medtronic Japan. MH received research funding from BIOTRONIK Japan, Medtronic Japan, Abbott Medical Japan, Japan Lifeline, Nihon Kohden and Boston Scientific Japan, and received speaker honoraria from Nippon Boehringer Ingelheim, Daiichi‐Sankyo, Bristol‐Meyers Squibb, Medtronic Japan, Japan Lifeline, and Abbott Medical Japan. MM received speaker honoraria from Medtronic Japan and Boston Scientific Japan. WS received grants from Daiichi Sankyo and Nippon Boehringer Ingelheim, and received remuneration for lectures, presentations, speakers bureaus, manuscript writing or educational events from Daiichi Sankyo, Nippon Boehringer Ingelheim, Bristol‐Meyers Squibb, K.K., Bayer Yakuhin, Pfizer, Ono Pharmaceutical and Medtronic Japan. KKu received speaker honoraria from Daiichi‐Sankyo, Ltd., Bayer Yakuhin, and Medtronic Japan, and received research grants from Medtronic Japan, HITACHI, and JSR. KI received speaker honoraria from Medtronic Japan and BIOTRONIK Japan. TH received speaker honoraria from Medtronic Japan, BIOTRONIK Japan and Bayer Yakuhin. IN has received speaking honoraria from Medtronic Japan. MS is affiliated with an endowed division supported by BIOTRONIK Japan, Boston Scientific Japan K.K, Medtronic Japan and Abbott Medical Japan. SHi is affiliated with an endowed division supported by BIOTRONIK Japan, Boston Scientific Japan K.K., Medtronic Japan and Abbott Medical Japan. IM received speaker honoraria from Daiichi‐Sankyo and Abbott Medical Japan. RKa received speaker honoraria from Daiichi‐Sankyo and Medtronic Japan, and received research grants from Boston Scientific Japan and Abbott Medical Japan. YIked received speaker honoraria from Bayer Yakuhin and Medtronic Japan. KKa has received research grants from Omron Healthcare Co., Ltd., A&D Co., Ltd., and Fukuda Denshi Co., Ltd. SS is affiliated with an endowed division supported by BIOTRONIK Japan, and received speaker honoraria from Medtronic Japan, Abbott Medical Japan, BIOTRONIK Japan, Boston Scientific Japan, and Japan Lifeline. YKo received speaker honoraria from Daiichi‐Sankyo, Bayer, Abbott Medical Japan, BIOTRONIK Japan, Boston Scientific Japan, Japan Lifeline, and received research funding from Daiichi‐Sankyo. KM has received honoraria for Chugai Pharmaceutical, AstraZeneca, Taiho Pharmaceutical, MSD, Kyowa Kirin, Yakult Pharmaceutical, and Boehringer Ingelheim. Other authors have no conflict of interest.

## FUNDING INFORMATION

This work is being funded in part by Medtronic Japan Co., Ltd. and Boston Scientific Corporation.

## ETHICS STATEMENT

It conforms to the Declaration of Helsinki and the Ethical Guidelines for Clinical Studies issued by the Ministry of Health, Labour and Welfare, Japan. This study protocol has been approved by the Institutional Review Board (IRB) of Nihon University Itabashi Hospital, Clinical Research Judging Committee (IRB no: RK‐221213‐1) and the participating hospitals' IRBs if required. Results of the study will be published in one or more peer‐reviewed journals.

## DECLARATIONS

*Approval of the Research Protocol*: This study protocol has been approved by the Institutional Review Board (IRB) of Nihon University Itabashi Hospital, Clinical Research Judging Committee (IRB no: RK‐221213‐1) and the participating hospitals' IRBs if required. *Informed Consent*: All study participants will provide written informed consent. *Registry and the Registration No. of the Study/Trial*: The study was registered with the UMIN Clinical Trials Registry (UMIN000050180). *Animal Studies*: N/A.

## PATIENT CONSENT STATEMENT

All study participants will provide written informed consent and may withdraw their consent at any time.

## CLINICAL TRIAL REGISTRATION

The study is registered with the UMIN Clinical Trials Registry (UMIN000050180).

## Supporting information


Figure S1.

## Data Availability

Neither patients nor members of the public have been or will be involved in the design of the study, its planning or the data collection or data analysis.
