# Supplementary material for: Multicenter prospective observational study to clarify the current status and clinical outcome in Japanese patients who have an indication for implantable cardioverter defibrillator (ICD) or wearable cardioverter defibrillator (WCD) (TRANSITION JAPAN‐ICD/WCD study): Rationale and design of a prospective, multicenter, observational, comparative study
Source: J Arrhythm. 2024 Mar 27;40(3):423–33. doi: 10.1002/joa3.13028 (PMC11199808; doi:10.1002/joa3.13028)

Transition-Japan ICD/WCD Study

Location	Participating Institutions (n=31)
Tokyo (n=10)	Nihon University Itabashi Hospital
	Nihon University Hospital
	Juntendo University Hospital
	Tokyo Rinkai Hospital
	Nippon Medical School Hospital
	Tokyo Metropolitan Hiroo Hospital
	Juntendo University Nerima Hospital
	Tokyo Women's Medical University Hospital
	Showa University Hospital
	Showa University Koto Toyosu Hospital
Kanagawa (n=3)	Kitasato University Hospital
	Shonan-Kamakura General Hospital
	St. Marianna University School of Medicine Hospital
Saitama (n=3)	Kawaguchi Municipal Medical Center
	Dokkyo Medical University Saitama Medical Center
	Saitama Medical University International Medical Center
Chiba (n=1)	Chiba University Hospital
Tochigi (n=1)	Jichi Medical University Hospital
Ishikawa (n=2)	Kanazawa University Hospital
	National Hospital Organization Kanazawa Medical Center
Aichi (n=1)	Fujita Health University Hospital
Hyogo (n=1)	Kobe University Hospital
Osaka (n=1)	National Cerebral and Cardiovascular Center
Gifu (n=1)	Ogaki Municipal Hospital
Yamaguchi (n=1)	Yamaguchi Prefectural Grand Medical Center

**Miyagi (n=1)**	Sendai Kousei Hospital
**Yamagata (n=1)**	Yamagata University Hospital
**Aomori (n=1)**	Hirosaki University Hospital
**Fukuoka (n=1)**	Shinkoga Hospital
**Kumamoto (n=1)**	Saiseikai Kumamoto Hospital
**Kagoshima (n=1)**	Kagoshima University Hospital

Statistical analysis

**Fukuoka (n=1)**	Biostatistics Center, Kurume University

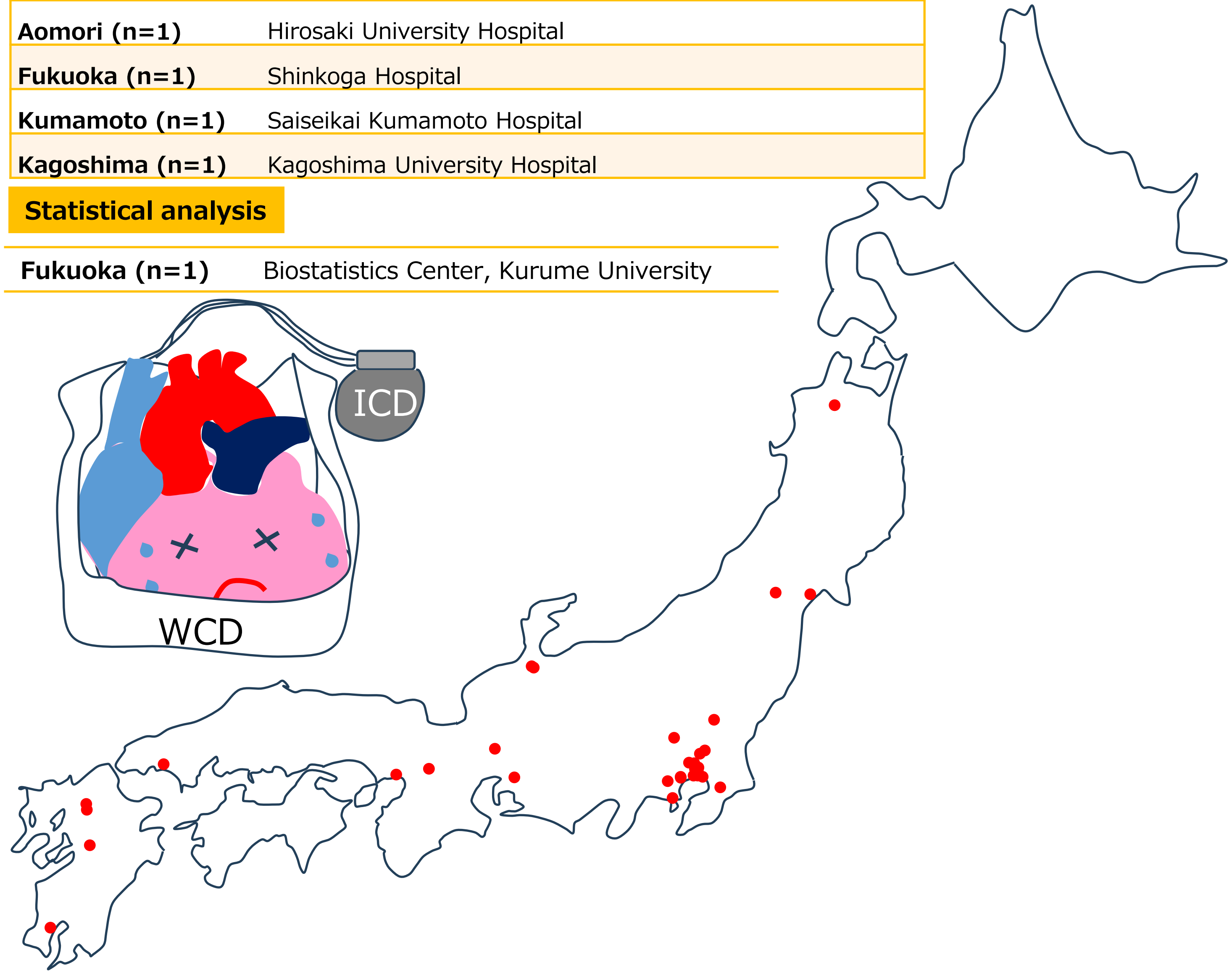

Supplement: Supplementary file 1 — Figure S1. [file JOA3-40-423-s001.pdf]
